# Process Optimization of *Sargassum fusiforme* Polyphenols and Their Cytoprotective Effects Against Oxidative and Inflammatory Injury, with Potential Signaling Correlates

**DOI:** 10.3390/foods15162821

**Published:** 2026-08-13

**Authors:** Le Li, Zhiqing Liu, Siting Wang, Quanhong Li

**Affiliations:** 1 School of Environmental and Quality Testing, Chongqing Chemical Industry Vocational College, No. 2009, East Bodhi Road, Changshou District, Chongqing 401228, China; 2 College of Food Science & Nutritional Engineering, China Agricultural University, No. 17 Tsinghua Dong Road, Beijing 100083, China; 18278289320@163.com; 3 School of Food Science and Engineering, South China University of Technology, No. 381 Wushan Road, Guangzhou 510640, China; 18278289375@163.com

**Keywords:** ultrasound-assisted extraction, response surface methodology, RAW 264.7 macrophages, endogenous antioxidant enzymes, inflammatory cytokine regulation

## Abstract

The development of marine-derived bioactive natural products has long been constrained by the dual bottlenecks of low extraction efficiency and unclear target mechanisms. In this study, single-factor experiments integrated with response surface methodology were employed to systematically establish an ultrasound-assisted high-efficiency extraction system for *Sargassum fusiforme* polyphenols (SP). The optimal extraction was achieved under the following conditions: ethanol concentration of 50%, liquid-to-solid ratio of 40 mL/g, ultrasonic power of 150 W, temperature of 50 °C, and extraction time of 60 min. Under these conditions, the experimentally obtained SP yield reached 38.91 ± 0.82 mg/g, showing no significant deviation from the model-predicted value (*p* > 0.05), thereby validating the reliability of the established process. In functional assessments, SP exhibited a mitigating effect on oxidative stress and inflammatory injury in cultured RAW 264.7 macrophages. In cellular assays, SP treatment was associated with elevated activities of endogenous antioxidant enzymes (SOD, GSH-Px and CAT), alongside reduced levels of pro-inflammatory mediators (NO, TNF-α, IL-1β, PGE_2_ and IL-6) and an increased level of IL-10 in LPS-stimulated macrophages. Collectively, this study establishes an efficient laboratory-scale extraction protocol for SP and presents in vitro evidence for its cytoprotective effects. These findings provide an empirical basis for future investigations into the bioactive potential of this marine polyphenol.

## 1. Introduction

Redox imbalance and inflammatory responses are core pathophysiological processes that occur in organisms in response to internal and external environmental stimuli [1,2]. These two processes are reciprocally coupled and act synergistically through a complex regulatory network centered on key nodes, including reactive oxygen species (ROS) and various pro-inflammatory mediators. Exogenous oxidative insults (e.g., hydrogen peroxide exposure) or endogenous inflammatory signals (e.g., lipopolysaccharide stimulation) can disrupt cellular redox homeostasis, thereby activating multiple signal transduction cascades, among which the Nrf2/ARE, NF-κB, and MAPK pathways represent the most canonical and critical regulatory axes [3,4]. Sustained activation or dysregulation of these signaling pathways has been extensively implicated in the initiation and progression of various chronic diseases, including neurodegenerative disorders, metabolic syndromes, cardiovascular diseases, and malignancies [5].

Currently, clinical strategies for intervening in oxidative stress and inflammatory responses primarily include synthetic antioxidants (e.g., N-acetylcysteine), non-steroidal anti-inflammatory drugs, and biological agents [5,6]. Although these agents exhibit definitive efficacy in alleviating acute inflammation or oxidative injury, their long-term or repeated use is frequently accompanied by notable adverse effects, such as gastrointestinal lesions, hepatorenal impairment, and immunosuppression [6]. Moreover, inconsistent drug responses or even drug resistance due to individual variability further constrain their clinical applicability [7]. Consequently, the development of alternative intervention strategies that combine multi-target synergistic regulatory capacity with high biosafety has become a critical scientific issue in this field that urgently needs to be addressed.

Natural polyphenols are a class of important secondary metabolites ubiquitously present in plants, structurally characterized by one or more aromatic rings bearing polyhydroxy substituents, and are mainly categorized into phenolic acids, flavonoids, tannins, and stilbenes [8]. Compared with synthetic antioxidants, natural polyphenols generally exhibit superior biosafety and broader-spectrum bioactivities, and their multi-target regulatory properties confer unique advantages in maintaining redox homeostasis and modulating inflammatory responses [9]. Therefore, screening for highly efficacious and low-toxicity polyphenolic active components from natural resources has emerged as a major research direction for the development of novel antioxidant and anti-inflammatory functional agents.

*Sargassum fusiforme* (*S. fusiforme*), a common brown alga belonging to the family *Sargassaceae*, is widely distributed along the coastal regions of East Asia, including China, Japan, and Korea [10]. The polyphenolic compounds present in this alga mainly include phloroglucinol derivatives, flavonoids, and phenolic acids [11]. However, at present, *S. fusiforme* resources are predominantly utilized for primary processing or direct consumption, whereas its abundant polyphenolic bioactive components remain inadequately exploited and valorized, resulting in an underutilization of its bioresource potential. Furthermore, existing extraction processes still suffer from issues such as low extraction efficiency and susceptibility of active components to degradation, which severely impede their further application in functional foods and pharmaceuticals.

To address the aforementioned challenges, this study aims to systematically optimize the extraction process of SP and to thoroughly elucidate the molecular mechanisms underlying their antioxidant and anti-inflammatory activities. Specifically, response surface methodology (RSM) is first employed to optimize the extraction conditions, with the goal of significantly enhancing polyphenol yield and extraction efficiency. On this basis, H_2_O_2_-induced oxidative stress models and lipopolysaccharide (LPS)-stimulated RAW 264.7 macrophage inflammatory models are established to systematically evaluate the cytoprotective effects of SPs against cellular injury, and to further dissect their regulatory mechanisms on three key signaling pathways, namely Nrf2/ARE, MAPK, and MyD88/NF-κB. This study is intended to provide a theoretical foundation for the functional evaluation of SF as novel natural antioxidant and anti-inflammatory bioactive factors, and to lay the experimental groundwork for their subsequent industrial development and application.

## 2. Materials and Methods

### 2.1. Materials and Reagents

Mature *S. fusiforme* was collected from Minhou County, Fuzhou, Fujian Province, China. Fresh thalli were freeze-dried for 72 h (Scientz-18N, Ningbo Scientz Biotechnology Co., Ltd., Ningbo, China), ground into powder, and passed through an 80-mesh sieve before extraction.

RAW 264.7 murine macrophages were obtained from Procell Life Science & Technology Co., Ltd. (Wuhan, China) and maintained in high-glucose Dulbecco’s modified Eagle’s medium (DMEM; Cytiva, Marlborough, MA, USA) supplemented with 10% (*v*/*v*) fetal bovine serum (Gibco, Grand Island, NY, USA) and 1% (*v*/*v*) penicillin–streptomycin at 37 °C in a humidified atmosphere containing 5% CO_2_.

Unless otherwise indicated, LPS, MTT, DMSO, DPPH, ABTS, gallic acid, H_2_O_2_, Pro-Prep lysis buffer, protease/phosphatase inhibitor cocktail and HRP-conjugated secondary antibodies were purchased from Sigma-Aldrich (St. Louis, MO, USA). TRIzol reagent was obtained from Thermo Fisher Scientific (Waltham, MA, USA), and Griess reagent was purchased from Beyotime Biotechnology (Shanghai, China). Kits for cDNA synthesis, quantitative real-time PCR, ELISA, and enzymatic activity assays were obtained from Nanjing Jiancheng Bioengineering Institute (Nanjing, China).

### 2.2. Single-Factor Experiments

Preliminary experiments were conducted to evaluate the effects of ethanol concentration (40–80%), ultrasonic power (90–210 W), liquid-to-solid ratio (30–50 mL g^−1^), extraction temperature (40–80 °C), and extraction time (30–150 min) on the extraction of SP. Each parameter was varied individually while the remaining conditions were held constant. Polyphenol yield was used as the response to determine the experimental range for subsequent optimization.

### 2.3. RSM Experimental Design

RSM was applied to optimize the extraction process based on the results of the single-factor experiments [7]. Extraction temperature and extraction time were fixed at 50 °C and 60 min, respectively, to minimize polyphenol degradation while maintaining extraction efficiency. A central composite design (CCD) generated using Design-Expert (v8.0, Stat-Ease) was employed to optimize ethanol concentration (A), liquid-to-solid ratio (B), and ultrasonic power (C). Polyphenol yield (Y) was selected as the response variable. The experimental design consisted of 17 runs, including replicated centre points for estimation of pure error and evaluation of model fit (Table S1).

### 2.4. SP Extraction, Purification, and LC-MS/MS Analysis

Polyphenols were extracted and purified as previously described by Wang et al. [8,11]. LC–MS/MS analysis was performed using an Agilent 1290 Infinity II UHPLC system coupled to an Agilent 6470 triple quadrupole mass spectrometer (Agilent Technologies, Santa Clara, CA, USA). Chromatographic separation was achieved on an Eclipse Plus C18 column (2.1 × 100 mm, 1.8 μm) maintained at 40 °C using a binary gradient of 0.1% formic acid in water (A) and acetonitrile (B) at a flow rate of 0.3 mL min^−1^ [8]. The injection volume was 2 μL. Mass spectra were acquired in negative electrospray ionization mode over an *m*/*z* range of 100–1500 [8,11].

### 2.5. Evaluation of Antioxidant Activity

#### 2.5.1. Determination of DPPH Radical Scavenging Capacity

DPPH radical scavenging activity was assessed following the method of Wu et al. [12] with minor modifications. Briefly, 100 µL of DPPH methanolic solution (100 µg/mL) was mixed with 100 µL of sample solutions at various concentrations in a 96-well plate. The mixture was incubated in the dark for 6 min, and the absorbance was recorded at 734 nm using a BioTek Epoch microplate reader (BioTek, Shoreline, WA, USA). A control consisting of 100 µL of DPPH solution and 100 µL of solvent was included. The radical scavenging capacity was expressed as Trolox equivalent antioxidant capacity (TEAC, mg TE/100 mL). To determine the antioxidant activity of individual polyphenolic compounds present in sugarcane vinegar, serial dilutions of each standard compound were prepared to obtain absorbance values within the linear range (0.2–0.8), and assayed as described above. IC_50_ values (µg/mL) were subsequently calculated by nonlinear regression analysis to quantify the antioxidant potency of each compound.

#### 2.5.2. Determination of ABTS Radical Scavenging Capacity

ABTS radical scavenging activity was determined according to the method of Wu et al. [12]. In brief, 150 µL of ABTS working solution was mixed with 50 µL of sample solutions at varying concentrations in a 96-well plate. The reaction mixture was incubated in the dark for 6 min, and the absorbance was measured at 734 nm using a BioTek Epoch microplate reader (BioTek, Winooski, VT, USA). Results were expressed as Trolox equivalent antioxidant capacity (TEAC, mg TE/100 mL).

#### 2.5.3. Cell Line, Culture Conditions and Assessment of Cell Viability

RAW 264.7 macrophages (ATCC, Manassas, VA, USA) were seeded in 96-well plates at a density of 2 × 10^5^ cells per well and cultured overnight at 37 °C in a humidified atmosphere containing 5% CO_2_. Cells were then treated with SP at graded concentrations (0.001–0.160 mg/mL), dissolved in sterile phosphate-buffered saline (PBS, pH 7.4), for 24 h. The final concentration of PBS in the culture medium did not exceed 0.1% (*v*/*v*) in all treatments. Cell viability was assessed using the MTT assay. Briefly, cells were incubated with MTT solution (0.5 mg mL^−1^) for 1 h at 37 °C. The resulting formazan crystals were dissolved in dimethyl sulfoxide (DMSO), and absorbance was measured at 570 nm using a BioTek Epoch microplate reader (BioTek, Winooski, VT, USA). Cell viability was normalized to that of untreated control cells (set as 100%). All experiments were performed with three independent biological replicates (n = 3).

#### 2.5.4. Intracellular Oxidative Stress Assessment

Intracellular reactive oxygen species (ROS) levels were quantified using the DCFH-DA fluorescent probe. RAW 264.7 macrophages were seeded in 96-well plates (2 × 10^5^ cells per well) and pretreated with SP (0.004 mg/mL, in PBS) for 2 h, followed by exposure to H_2_O_2_ (0.5 mM) for 2 h at 37 °C [8]. After treatment, cells were incubated with DCFH-DA (10 µM) in serum-free medium for 30 min at 37 °C in the dark. Fluorescence intensity was recorded at excitation/emission wavelengths of 488/525 nm using a microplate reader. ROS levels were expressed as fold change relative to the H_2_O_2_-treated control group (set as 1.0).

For the determination of glutathione homeostasis and lipid peroxidation, intracellular reduced glutathione (GSH), oxidized glutathione (GSSG), and malondialdehyde (MDA) were quantified using commercial assay kits (Beyotime Biotechnology, Shanghai, China; or Nanjing Jiancheng Bioengineering Institute, Nanjing, China) according to the manufacturers’ protocols. All values were normalized to total protein content determined by the bicinchoninic acid (BCA) method.

#### 2.5.5. Quantitative Real-Time PCR

RAW 264.7 macrophages were seeded in 6-well plates (1 × 10^6^ cells per well) and pretreated with SP (0.004 mg/mL, in PBS) for 2 h, followed by exposure to H_2_O_2_ (0.5 mM) for 2 h. Total RNA was extracted using TRIzol reagent (Invitrogen, Carlsbad, CA, USA) according to the manufacturer’s instructions, and reverse-transcribed into cDNA using the SensiFAST™ cDNA Synthesis Kit (Meridian Bioscience, Cincinnati, OH, USA) [8]. Quantitative real-time PCR was performed using the SensiFAST™ SYBR No-ROX Kit (Meridian Bioscience) on a QuantStudio™ 1 Real-Time PCR System (Applied Biosystems, Foster City, CA, USA). The thermal cycling protocol consisted of an initial denaturation at 95 °C for 2 min, followed by 40 cycles of 95 °C for 5 s and 60 °C for 30 s. Relative transcript abundance was calculated using the 2^−ΔΔCt^ method, with β-actin as the internal reference gene [13]. Target gene expression was normalized to β-actin and expressed as fold change relative to the H_2_O_2_-treated control group. Primer sequences are listed in Supplementary Table S2.

#### 2.5.6. Antioxidant Enzyme Activities

The activities of superoxide dismutase (SOD), glutathione peroxidase (GSH-Px), and catalase (CAT) were determined using commercial assay kits (Beyotime Biotechnology, Shanghai, China) strictly according to the manufacturers’ instructions [7]. Briefly, RAW 264.7 macrophages were seeded in 6-well plates (1 × 10^6^ cells per well), pretreated with SP (0.004 mg/mL, in PBS) for 2 h, and stimulated with H_2_O_2_ (0.5 mM) for 2 h. Cells were harvested, lysed by sonication in cold PBS, and centrifuged at 12,000× *g* for 15 min at 4 °C. Supernatants were collected for enzyme activity assays. SOD activity was measured by the WST-8 method; GSH-Px activity was determined by measuring the decrease in NADPH absorbance at 340 nm; CAT activity was assessed by monitoring the decomposition of H_2_O_2_ at 240 nm. All enzyme activities were normalized to total protein content, determined using the BCA method, and expressed as U/mg protein.

#### 2.5.7. Western Blotting

RAW 264.7 macrophages were seeded in 6-well plates (1 × 10^6^ cells per well) and pretreated with SP (0.004 mg/mL, in PBS) for 2 h, followed by stimulation with H_2_O_2_ (0.5 mM) for 2 h. Whole-cell lysates were prepared using Pro-Prep™ lysis buffer (iNtRON Biotechnology, Seongnam, Republic of Korea) supplemented with protease inhibitor cocktail and phosphatase inhibitors (Roche, Basel, Switzerland). Protein concentrations were determined using the BCA method. Equal amounts of protein (20 µg per lane) were separated by SDS-PAGE (12% resolving gel) and transferred onto PVDF membranes (Millipore, Billerica, MA, USA). Membranes were blocked with 5% (*w*/*v*) non-fat milk in Tris-buffered saline containing 0.1% Tween-20 (TBST) for 1 h at room temperature, followed by overnight incubation at 4 °C with primary antibodies against NRF2 (1:1000), KEAP1 (1:1000), HMOX1 (1:1000), NQO1 (1:1000), and β-actin (1:5000). After washing with TBST, membranes were incubated with horseradish peroxidase (HRP)-conjugated secondary antibodies (1:5000) for 1 h at room temperature. Immunoreactive bands were visualized using enhanced chemiluminescence (ECL) substrate (Millipore) and captured on a ChemiDoc™ Imaging System (Bio-Rad, Hercules, CA, USA). Band densities were quantified using ImageJ software (version 1.54p, NIH, Bethesda, MD, USA). All target protein levels were normalized to the corresponding β-actin loading control and expressed as fold change relative to the H_2_O_2_-treated control group.

### 2.6. Evaluation of Anti-Inflammatory Activity

#### 2.6.1. Nitric Oxide (NO) Production

Nitric oxide production was quantified using the Griess assay. RAW 264.7 macrophages were seeded in 96-well plates (2 × 10^5^ cells per well) and pretreated with SP (0.004 mg/mL, in PBS) for 2 h, followed by stimulation with lipopolysaccharide (LPS, 1 µg mL^−1^, from Escherichia coli O111:B4; Sigma-Aldrich, St. Louis, MO, USA) for 24 h [7]. Cell culture supernatants were collected and mixed with an equal volume of Griess reagent (1% sulfanilamide in 5% H_3_PO_4_ and 0.1% N-(1-naphthyl) ethylenediamine dihydrochloride). After incubation for 15 min at room temperature in the dark, absorbance was measured at 540 nm using a microplate reader. NO concentration was calculated from a sodium nitrite (NaNO_2_) standard curve (0–100 µM) and normalized to the LPS-treated control group.

#### 2.6.2. Quantitative Real-Time PCR

RAW 264.7 macrophages were seeded in 6-well plates (1 × 10^6^ cells per well) and pretreated with SP (0.004 mg/mL, in PBS) for 2 h, followed by stimulation with LPS (1 µg mL^−1^) for 24 h. Total RNA isolation, cDNA synthesis, and quantitative real-time PCR were performed as described in Section 2.5.5. Primer sequences for inflammatory cytokines (TNF-α, IL-1β, IL-6, IL-10) and MAPK/NF-κB signaling components are listed in Supplementary Table S3. Gene expression was normalized to β-actin and expressed as fold change relative to the LPS-treated control group using the 2^−ΔΔCt^ method.

#### 2.6.3. Enzyme-Linked Immunosorbent Assay

RAW 264.7 macrophages were seeded in 12-well plates (5 × 10^5^ cells per well) and pretreated with SP (0.004 mg/mL, in PBS) for 2 h, followed by stimulation with LPS (1 µg mL^−1^) for 24 h [7]. Cell culture supernatants were collected and centrifuged at 1000× *g* for 10 min at 4 °C to remove cellular debris. The levels of prostaglandin E_2_ (PGE_2_), interleukin-6 (IL-6), tumor necrosis factor-α (TNF-α), and interleukin-10 (IL-10) in the supernatants were determined using commercial ELISA kits (R&D Systems, Minneapolis, MN, USA; or eBioscience, San Diego, CA, USA) according to the manufacturers’ protocols [7]. All cytokine concentrations were calculated from standard curves and normalized to the LPS-treated control group.

#### 2.6.4. Western Blotting (Inflammatory Signaling)

RAW 264.7 macrophages were seeded in 6-well plates (1 × 10^6^ cells per well) and pretreated with SP (0.004 mg/mL, in PBS) for 2 h, followed by stimulation with LPS (1 µg mL^−1^) for 24 h [7]. Whole-cell lysates were prepared and protein concentrations determined as described in Section 2.5.7. Equal amounts of protein (20 µg per lane) were separated by SDS-PAGE and transferred onto PVDF membranes [7]. After blocking with 5% non-fat milk in TBST, membranes were incubated overnight at 4 °C with primary antibodies against MyD88 (1:1000), ERK1/2 (1:1000), phospho-ERK1/2 (Thr202/Tyr204, 1:1000), JNK (1:1000), phospho-JNK (Thr183/Tyr185, 1:1000), p38 (1:1000), phospho-p38 (Thr180/Tyr182, 1:1000), IκBα (1:1000), phospho-IκBα (Ser32/36, 1:1000), p65 (1:1000), phospho-p65 (Ser536, 1:1000), and β-actin (1:5000). All primary antibodies were obtained from Cell Signaling Technology (Danvers, MA, USA) or Abcam (Cambridge, UK). Following incubation with HRP-conjugated secondary antibodies (1:5000) for 1 h at room temperature, immunoreactive bands were visualized and quantified as described in Section 2.5.7. All target protein levels were normalized to the corresponding β-actin loading control and expressed as fold change relative to the LPS-treated control group.

### 2.7. Statistical Analysis

Statistical analyses were performed using SPSS (version 20.0, IBM, Armonk, NY, USA). Response surface methodology (RSM) was conducted using Design-Expert (version 10.0, Stat-Ease, Minneapolis, MN, USA). All data are presented as mean ± standard deviation (SD) from at least three independent biological replicates, with three technical replicates per biological replicate, unless otherwise stated in the figure legends. Group comparisons were evaluated by one-way analysis of variance (ANOVA) followed by Duncan’s multiple range test. The specific n values for each experiment are indicated in the corresponding figure legends, where n represents the number of biologically independent samples. Statistical significance was defined as *p* < 0.05. In all figures, significance levels are denoted as *p* < 0.05 (*), *p* < 0.01 (**), and *p* < 0.001 (***).

## 3. Results and Discussion

### 3.1. Optimal Extraction Conditions Achieved Through Single-Factor Experiments and RSM

**Figure 1:**
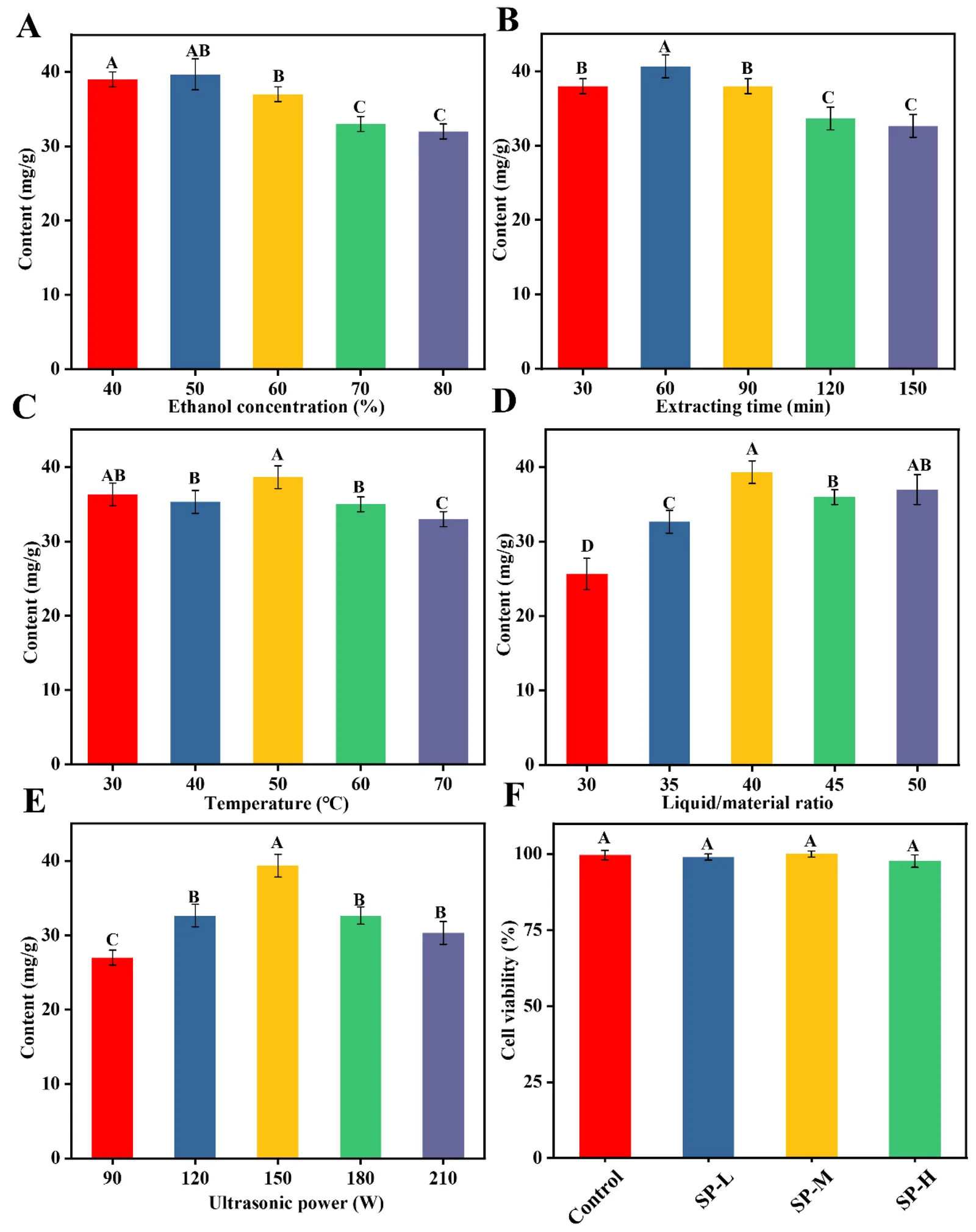
Single-factor optimization of ultrasound-assisted extraction parameters for polyphenols from *S. fusiforme* and cytotoxic evaluation in RAW 264.7 macrophages: (**A**) ethanol concentration, (**B**) extraction time, (**C**) temperature, (**D**) liquid-to-solid ratio, (**E**) ultrasonic power, (**F**) cell viability. The data are shown as mean ± SD (n = 3). Different uppercase letters (A–D) above the bars denote significant differences (*p* < 0.05) among treatments within the same experimental factor.

As shown in Figure 1A, the extraction yield of SP increased with increasing ethanol concentration, reached a maximum at 50%, and subsequently declined as the ethanol concentration increased further. Moderate ethanol concentrations are thought to enhance the disruption of hydrogen-bond interactions between polyphenols and cell wall components, thereby facilitating polyphenol release. In contrast, higher ethanol concentrations reduce solvent polarity, resulting in lower solubility of polar phenolic compounds while promoting the co-extraction of lipophilic constituents, such as chlorophyll, which may compromise extraction efficiency and interfere with polyphenol quantification [14,15]. Therefore, 50% ethanol was selected for subsequent experiments.

The effect of extraction time on polyphenol yield displayed a pattern of initial increase and subsequent decrease (Figure 1B), with the highest yield observed at 60 min, after which the yield declined. This phenomenon may be attributable to oxidative degradation or polymerization of polyphenols induced by the synergistic effects of prolonged heating and ultrasonication, as well as the continuous catalytic oxidation of phenolic substrates by oxidative enzymes released into the liquid phase [16]. Therefore, to balance extraction efficiency with minimal polyphenol degradation, 60 min was adopted as the optimal extraction time.

As illustrated in Figure 1C, the polyphenol yield from *S. fusiforme* increased with rising extraction temperature, peaking at 50 °C and then decreasing significantly above this threshold. Elevated temperatures accelerate mass transfer and reduce solvent viscosity, facilitating the release of polyphenols from the matrix; however, when the temperature exceeds a certain threshold, the phenolic hydroxyl groups in polyphenol molecules are susceptible to auto-oxidation or thermally induced cleavage, resulting in reduced yield [17]. Considering the critical influence of temperature on polyphenol stability, 50 °C was determined as the optimal extraction temperature.

As shown in Figure 1D, the polyphenol yield increased with the liquid-to-solid ratio and reached a maximum at 40 mL g^−1^, followed by a slight decline at higher ratios. A sufficient solvent volume enhances mass transfer and promotes the release of polyphenols into the extraction medium. However, excessive solvent volumes may reduce the concentration gradient between the solid and liquid phases, thereby lowering extraction efficiency under fixed extraction conditions [18]. Accordingly, a liquid-to-solid ratio of 40 mL g^−1^ was selected for subsequent experiments.

The influence of ultrasonic power on polyphenol yield from *S. fusiforme* exhibited a parabolic profile, with the highest yield achieved at 150 W (Figure 1E). Appropriate ultrasonic power enhances cavitation effects, disrupting cell wall structures and promoting intracellular polyphenol release; however, excessively high power may induce localized high temperatures and excessive accumulation of hydroxyl radicals generated during cavitation bubble collapse, which can attack the phenolic ring structures of polyphenol molecules and trigger oxidative degradation [19,20]. Considering the balance between equipment stability and extraction efficiency, extraction temperature and time were fixed at 50 °C and 60 min for subsequent response surface optimization, with ethanol concentration, liquid-to-solid ratio, and ultrasonic power selected as the three parameters for further optimization.

RSM was subsequently applied to optimize the extraction conditions. Analysis of variance indicated that ethanol concentration had the greatest effect on polyphenol yield, followed by the liquid-to-solid ratio and ultrasonic power. Three-dimensional response surface plots and interaction analyses revealed a significant interaction between ethanol concentration and liquid-to-solid ratio (Figure S1A–D; Tables S4 and S5), indicating that solvent composition and solvent volume jointly affected polyphenol extraction. The optimized extraction conditions predicted by the model were 50% ethanol, a liquid-to-solid ratio of 40 mL g^−1^, and an ultrasonic power of 150 W, corresponding to a predicted polyphenol yield of 39.23 mg g^−1^ dry weight. Validation experiments performed in triplicate under the optimized conditions produced an average yield of 38.91 ± 0.82 mg g^−1^, which did not differ significantly from the predicted value (*p* > 0.05). These results demonstrate the satisfactory predictive performance of the model and confirm the reproducibility of the optimized extraction conditions.

### 3.2. SP Samples Exhibited No Cytotoxicity at the Tested Concentrations

A total of 13 small-molecule compounds were identified in SP, comprising 11 fuhalol-type phlorotannins and two flavonoids, a composition consistent with that reported by Wang et al. [11] (Figure S2 and Table S6). To exclude non-specific factors that might confound the reported antioxidant and anti-inflammatory activities, the MTT assay was employed to investigate the effects of SPs on the viability of RAW 264.7 macrophages at various test concentrations. As shown in Figure 1F, in the concentration range from 0.02 to 0.06 mg/mL, cell viability in the SP-L, SP-M, and SP-H treatment groups consistently remained above 95%, with no statistically significant differences in comparison with the untreated group (*p* > 0.05). These findings indicate that the bioactive components in SP samples did not induce detectable cell membrane damage or metabolic dysfunction at the tested concentrations. Accordingly, cytotoxicity can be excluded as a confounding factor in subsequent evaluations of antioxidant and anti-inflammatory activities, thereby providing a reliable basis for accurately elucidating the molecular regulatory mechanisms of SP.

### 3.3. In Vitro Antioxidant Capacity of SP

**Figure 2:**
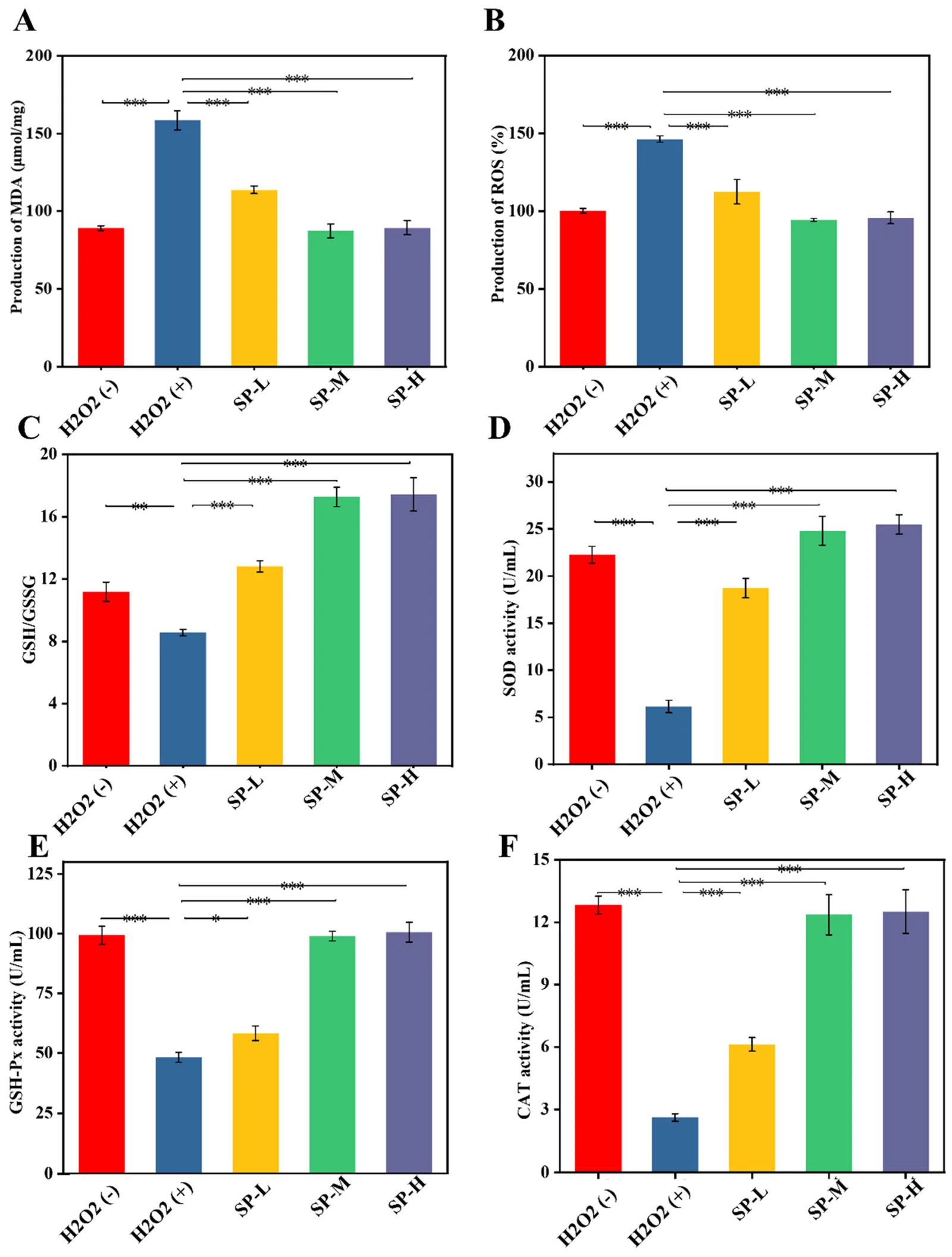
In vitro antioxidant capacity of SP in RAW 264.7 macrophages: (**A**) MDA, (**B**) ROS, (**C**) GSH/GSSG. Activities of (**D**) SOD, (**E**) GSH-Px, and (**F**) CAT. *p* < 0.05 (*), *p* < 0.01 (**), and *p* < 0.001 (***).

The antioxidant activity of SP was evaluated using chemical radical scavenging assays together with a cellular oxidative stress model. As presented in Table S7, SP exhibited a dose-dependent radical scavenging activity against both DPPH and ABTS radicals across the concentration range of 0.0002–0.0160 mg/mL. The IC_50_ values of SP were determined to be 0.00198 mg/mL (95% CI: 0.00172–0.00228 mg/mL) for DPPH and 0.0212 mg/mL (95% CI: 0.00185–0.00243 mg/mL) for ABTS, which were comparable to those of ascorbic acid (0.00145 and 0.00158 mg/mL, respectively). The high coefficients of determination (R^2^ > 0.99) confirmed the reliability of the dose–response model fitting. Notably, at the concentration of 0.0020 mg/mL, SP achieved approximately 50% inhibition in both assays, consistent with the calculated IC_50_ values. In H_2_O_2_-treated RAW 264.7 macrophages, SP pretreatment significantly reduced intracellular ROS accumulation and MDA production while restoring the GSH/GSSG ratio in a concentration-dependent manner (Figure 2A–C). These findings indicate that SP effectively alleviates oxidative stress both through direct radical scavenging and by preserving intracellular redox homeostasis, consistent with the reported antioxidant properties of polyphenolic compounds [21,22].

### 3.4. Protective Effects of SP Against Cellular Oxidative Stress

To further investigate the antioxidant mechanism of SP, the mRNA expression of key antioxidant enzymes was quantified in H_2_O_2_-stimulated RAW 264.7 macrophages. As shown in Figure S3A–D, H_2_O_2_ exposure markedly reduced the expression of SOD1, SOD2, CAT, and GSH-Px, whereas SP pretreatment significantly increased the transcript levels of all four genes (*p* < 0.05). This effect was concentration-dependent over the range of 0.02–0.04 mg mL^−1^, while no further increase was observed at concentrations above 0.04 mg mL^−1^ (*p* > 0.05). These results suggest that SP enhances the cellular antioxidant defense system by promoting the transcription of antioxidant enzyme genes, consistent with the established role of these enzymes in maintaining intracellular redox homeostasis [23,24].

At the functional level, the activities of three antioxidant enzymes, SOD, GSH-Px, and CAT, were further assessed. Figure 2D–F show that all three enzyme activities were evidently enhanced in H_2_O_2_-stimulated RAW 264.7 cells following SP pretreatment (*p* < 0.05), with trends highly consistent with the mRNA expression patterns. Notably, when the SP concentration exceeded 0.04 mg/mL, the increases in enzyme activities tended to saturate, further corroborating the dose-dependent characteristics of transcriptional regulation. Collectively, these results demonstrate that SPs can synergistically alleviate cellular oxidative injury via boosting the transcription of antioxidant enzyme genes and enhancing the corresponding enzyme activities.

### 3.5. Activation of the Nrf2/ARE Signaling Pathway in RAW 264.7 Cells by SP

**Figure 3:**
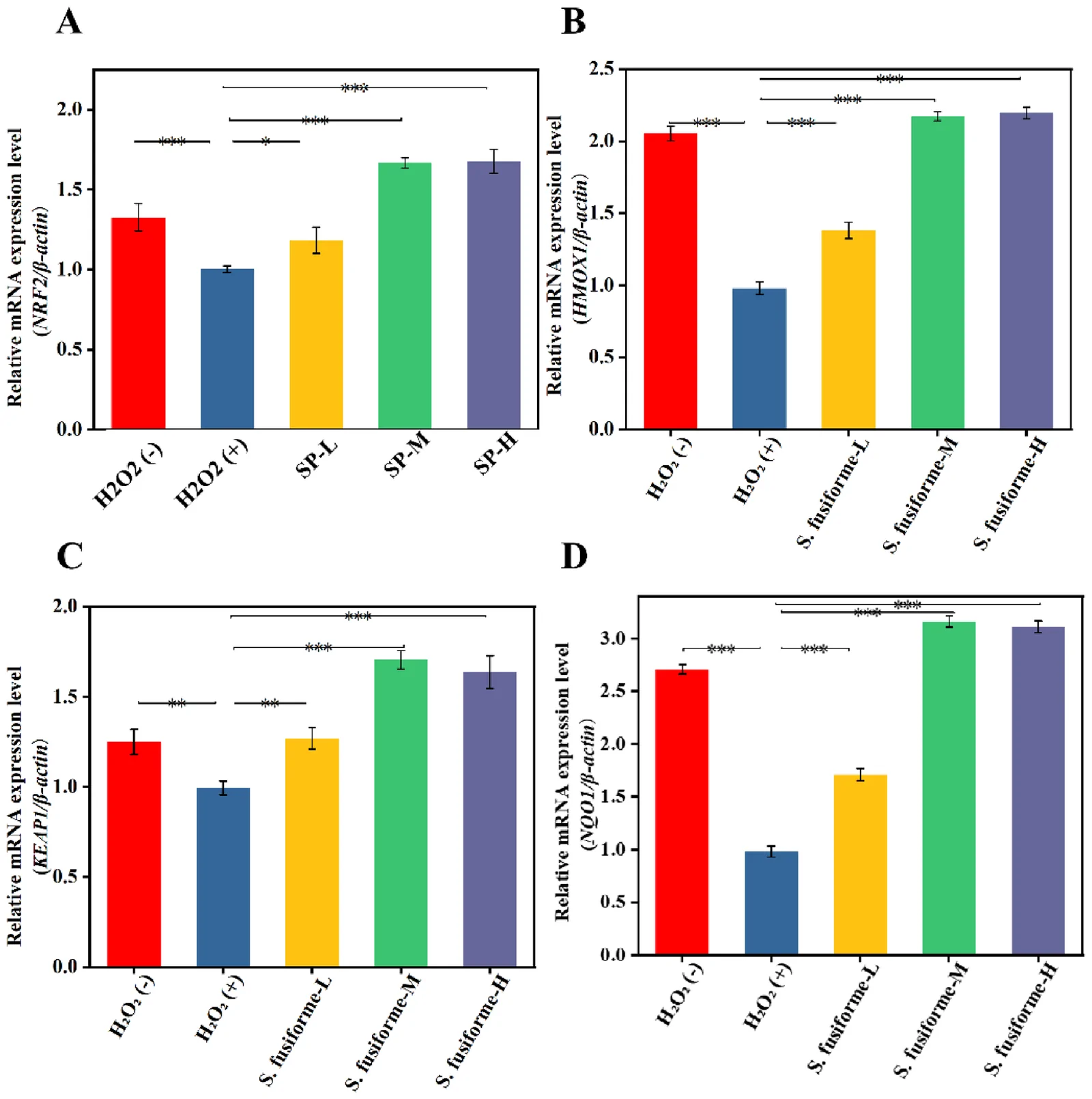
Effects of SP on the Nrf2/ARE signaling pathway in RAW 264.7 macrophages. Relative mRNA expression levels of (**A**) NRF2, (**B**) HMOX1, (**C**) KEAP1, and (**D**) NQO1. *p* < 0.05 (*), *p* < 0.01 (**), and *p* < 0.001 (***).

To investigate the molecular basis underlying the antioxidant effects of SP, the expression of genes associated with the Nrf2/ARE pathway was analyzed in H_2_O_2_-treated RAW 264.7 macrophages. As shown in Figure 3A–D, H_2_O_2_ treatment significantly increased the mRNA levels of NRF2, HMOX1, KEAP1, and NQO1 compared with the untreated control (*p* < 0.05). SP pretreatment further upregulated the expression of these genes (*p* < 0.05), with the greatest response observed at 0.04 mg mL^−1^. No significant increase was detected at higher SP concentrations (*p* > 0.05), indicating a plateau in the transcriptional response. These observations indicate that SP treatment was associated with enhanced transcription of Nrf2/ARE pathway-related genes, consistent with previous reports implicating this pathway in the cellular antioxidant response [25,26,27]. However, because transcriptional upregulation alone does not establish causative regulation, and because subcellular fractionation was not performed in this study to assess Nrf2 nuclear translocation, the mechanistic connection between SP and Nrf2/ARE pathway activation remains correlative at this stage.

Western blotting was performed to determine whether the transcriptional changes observed in the Nrf2/ARE pathway were accompanied by corresponding changes in protein expression. As shown in Figure S4A–D, H_2_O_2_ treatment significantly decreased the protein abundance of Nrf2, Keap1, HO-1, and NQO1 compared with the untreated control. SP pretreatment markedly increased the expression of these proteins in a concentration-dependent manner, consistent with the corresponding changes in mRNA expression. Collectively, these findings demonstrate a correlation between SP treatment and the upregulation of Nrf2/ARE pathway components at both the transcriptional and translational levels. It should be noted, however, that the present data do not provide direct evidence for a physical interaction between SP-derived compounds and KEAP1, nor do they establish a causal link between SP treatment and NRF2 activation. Direct mechanistic validation, such as assessment of NRF2 nuclear translocation via subcellular fractionation or immunofluorescence, evaluation of KEAP1–NRF2 interaction by co-immunoprecipitation, or genetic perturbation of NRF2 expression, is required to substantiate the involvement of this pathway in future studies.

### 3.6. Regulation of SP on LPS-Stimulated Inflammation in RAW 264.7 Cells

The antioxidant effects of SP appear to involve the Nrf2/ARE signalling pathway. In addition to regulating the expression of antioxidant genes, activated Nrf2 has been reported to suppress inflammatory responses through negative crosstalk with NF-κB signalling, suggesting a potential mechanistic link between the antioxidant and anti-inflammatory activities of SP [28]. The anti-inflammatory effects of SPs might involve crosstalk with the NF-κB pathway, potentially through elevation of antioxidant proteins such as HO-1, which can block IκBα phosphorylation and degradation. However, the proposed direct modification of phosphorylation sites on the NF-κB p65 subunit by SP is not experimentally validated in this study, as no phospho-proteomic or specific kinase activity assays were conducted. The anti-inflammatory effects of SPs may arise from direct modification of phosphorylation sites on the NF-κB p65 subunit, or through elevation of antioxidant proteins such as HO-1, which blocks IκBα phosphorylation and degradation, consequently inhibiting the translocation of NF-κB into the nucleus. To evaluate the regulation of SP on LPS-induced inflammatory responses in RAW 264.7 macrophages, key pro-inflammatory mediators (iNOS, COX-2), pro-inflammatory cytokines (IL-6, IL-1β, TNF-α), and the anti-inflammatory cytokine IL-10 were selected as representative indicators for detection according to reference [13]. qRT-PCR analysis demonstrated that LPS treatment substantially elevated the mRNA levels of all pro-inflammatory targets (*p* < 0.001) (Figure S5A–F). In contrast, SP pretreatment effectively suppressed the expression of pro-inflammatory mediators and cytokines while significantly enhancing IL-10 transcription, exhibiting a clear dose-dependent pattern. Notably, when the SP concentration was higher than 0.04 mg/mL, the expression levels of all inflammation-related genes plateaued, with no notable differences noted among treatment groups (*p* > 0.05). This saturation phenomenon may be attributed to the TLR4/NF-κB signaling axis reaching its maximum response threshold. Nevertheless, the alternative interpretation, involving saturation of binding sites on target proteins by active SP constituents, lacks direct experimental support from binding affinity assays and should therefore be considered a hypothesis rather than a confirmed mechanism [29,30].

**Figure 4:**
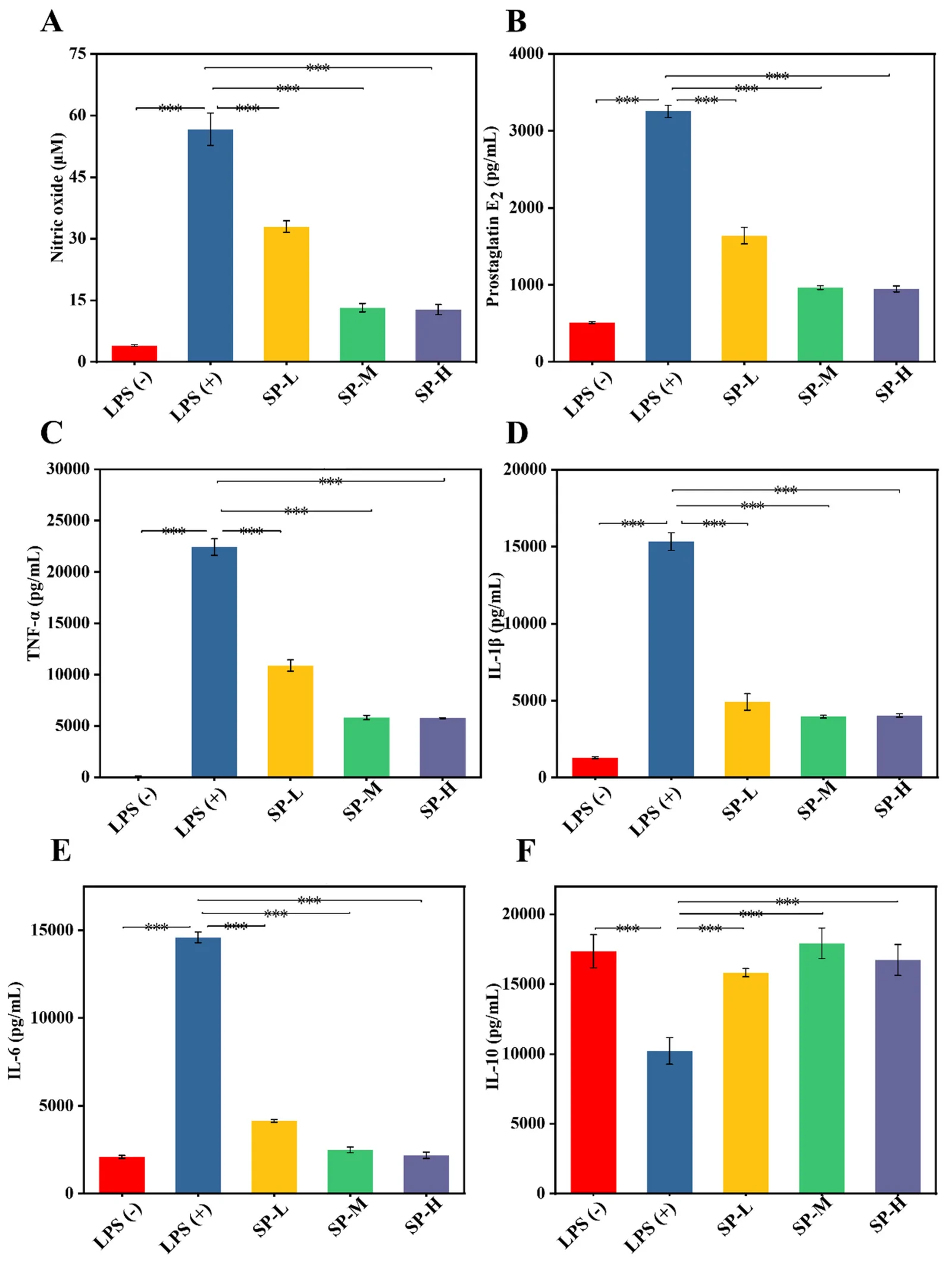
Inflammatory factor levels: (**A**) Nitric oxide (NO), (**B**) PGE_2_, (**C**) TNF-α, (**D**) IL-1β, (**E**) IL-6, and (**F**) IL-10. *p* < 0.001 (***).

To further assess the functional consequences of the transcriptional changes, the secretion of inflammatory mediators was quantified in LPS-stimulated RAW 264.7 macrophages. As shown in Figure 4A–E, LPS stimulation significantly increased the production of NO, PGE_2_, TNF-α, IL-1β, and IL-6 compared with the untreated control (*p* < 0.05). SP pretreatment markedly suppressed the secretion of these pro-inflammatory mediators in a concentration-dependent manner. The greatest inhibitory effect was observed in the SP-H (0.06 mg mL^−1^) group, although no significant difference was detected between the SP-H and SP-M (0.04 mg mL^−1^) groups (*p* > 0.05), consistent with the plateau observed in the corresponding gene expression analysis. In contrast, SP significantly increased the secretion of the anti-inflammatory cytokine IL-10 (*p* < 0.05; Figure 4F). Together, these findings demonstrate that SP attenuates LPS-induced inflammatory responses by suppressing the production of pro-inflammatory mediators while promoting the secretion of anti-inflammatory cytokines, consistent with the transcriptional changes observed above.

### 3.7. SP Suppresses LPS-Induced Activation of MAPK and MyD88/NF-κB Signaling in RAW 264.7 Cells

Previous findings demonstrated that SP treatment was associated with altered transcription of multiple inflammatory mediators, including iNOS, COX-2, IL-6, IL-1β, TNF-α, and IL-10, suggesting possible involvement of the MAPK and NF-κB signaling cascades in its anti-inflammatory effects. To further explore the molecular correlates underlying SP-associated inflammation suppression, RT-qPCR analysis was performed to evaluate the expression of representative genes associated with these signaling pathways in LPS-challenged RAW 264.7 macrophages. As shown in Figure S6A–C, LPS exposure significantly increased the mRNA levels of ERK1/2, JNK, and p38 relative to the untreated control (*p* < 0.01), corroborating the canonical activation of the MAPK pathway downstream of TLR4 signaling [31]. SP pretreatment significantly reduced the mRNA levels of these genes in a concentration-dependent manner, with the greatest suppression observed at 0.04 mg mL^−1^. Notably, when the SP concentration exceeded 0.04 mg mL^−1^, no further reduction in MAPK gene expression was observed (*p* > 0.05), suggesting a saturable transcriptional response. It should be emphasized, however, that the present data do not establish whether this saturation arises from competitive binding to TLR4, direct interference with the TLR4–MyD88 interaction, or other mechanisms, as we did not perform molecular docking, binding kinetics, co-immunoprecipitation, or site-directed mutagenesis experiments to address this question [32].

In the NF-κB signaling cascade, LPS treatment led to significantly elevated mRNA levels of MyD88, IκB-α, and p65 (Figure S6D–F), indicating effective activation of the pathway. SP pretreatment significantly suppressed the transcriptional activation of these genes, with the SP-H (0.06 mg/mL) group exhibiting the strongest inhibitory effect; however, this effect was not significantly different from that of the SP-M (0.04 mg/mL) group (*p* > 0.05). These observations indicate that SP treatment correlated with reduced transcription of NF-κB pathway components. However, the inference that this suppression occurs via direct interference with the TLR4–MD2 complex or MyD88-dependent signaling is not experimentally substantiated by our current dataset. In the absence of direct molecular docking, binding affinity measurements, or protein–protein interaction assays, any such model should be considered speculative and reserved for future investigation [33,34].

**Figure 5:**
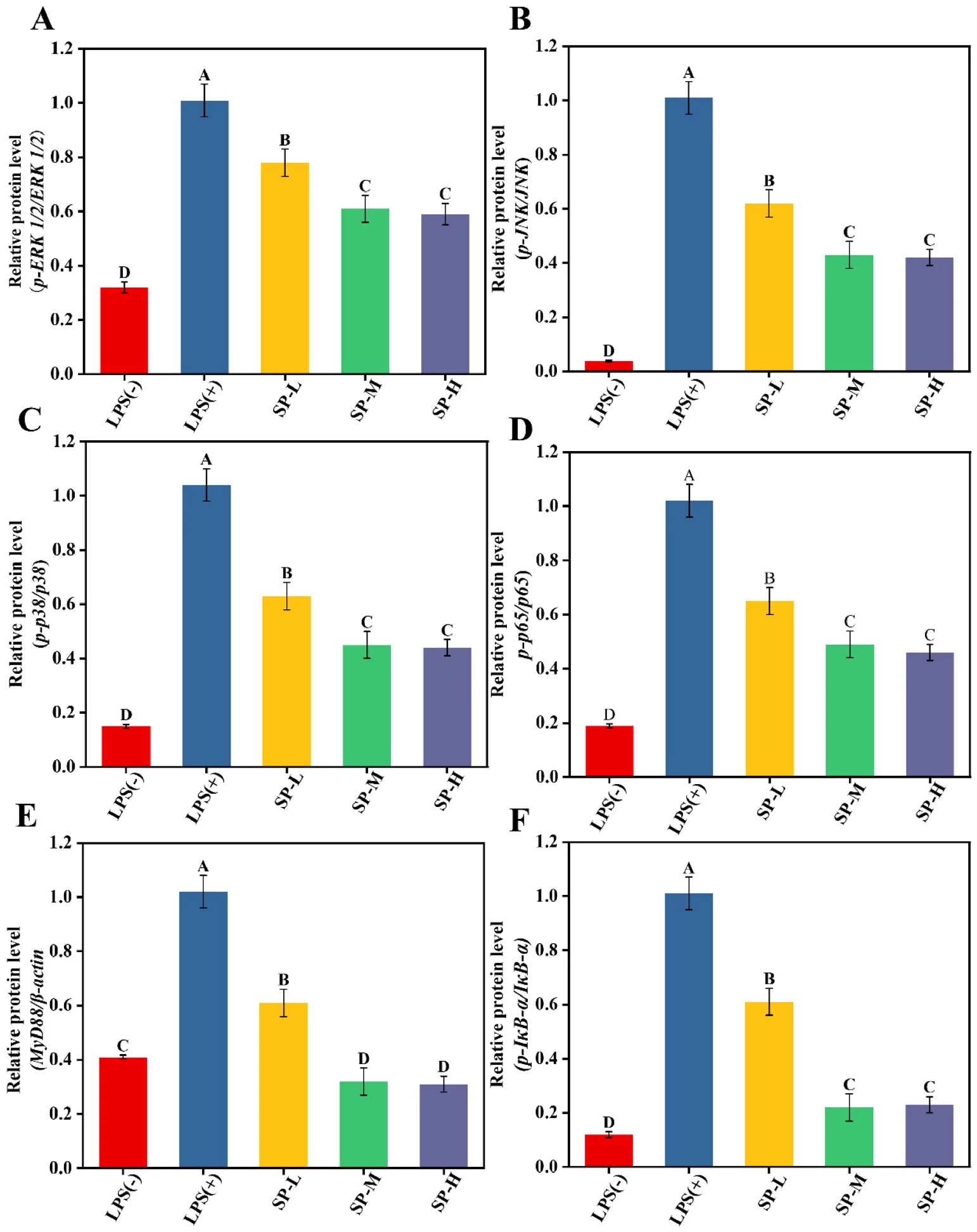
Effects of SP on LPS-induced activation of MAPK and MyD88/NF-κB signaling pathways in RAW 264.7 macrophages. The relative protein expression level of (**A**) p-ERK 1/2/ERK 1/2, (**B**) p-JNK/JNK, (**C**) p-p38/p38, (**D**) p-p65/p65, (**E**) MyD88/β-actin, and (**F**) p-IκB-α/IκB-α. The data are shown as mean ± SD (n = 3). Different uppercase letters (A–D) above the bars denote significant differences (*p* < 0.05) among treatments within the same experimental factor.

To verify whether the changes observed at the transcriptional level were recapitulated at the protein level, and to further delineate the regulatory effects of SP on inflammation-related signaling pathways, Western blot analysis was employed to examine the expression of proteins involved in the MAPK and NF-κB pathways in LPS-stimulated RAW 264.7 cells. LPS treatment notably elevated the protein levels of phosphorylated ERK1/2, JNK, and p38, whereas SP pretreatment significantly reduced their phosphorylation in a concentration-dependent manner (Figure 5A–C). In the NF-κB pathway, SP similarly and significantly reduced the LPS-induced protein levels of MYD88, phosphorylated IκB-α, and phosphorylated p65 (Figure 5D–F). Consistent with the results observed in the MAPK cascade, no statistically significant differences were observed between the SP-M (0.04 mg/mL) and SP-H (0.06 mg/mL) treatment groups in terms of inhibiting protein phosphorylation (*p* > 0.05), further corroborating the dose-saturation characteristics of SP-associated modulation of TLR4-triggered inflammatory signaling pathways. Taken together, the transcriptional and translational results demonstrate that SP treatment correlates with reduced activation of MAPK and NF-κB signaling components in LPS-stimulated macrophages. However, the present findings do not establish that SP directly targets TLR4, MD2, MYD88, or any specific upstream receptor component. The observed effects are consistent with, but do not prove, modulation at or near the receptor level. Direct evidence, such as surface plasmon resonance-based binding assays, co-immunoprecipitation of TLR4–MYD88 complexes, or CRISPR-mediated knockdown of pathway components, is required to validate the underlying mechanisms. These results provide an in vitro experimental basis for the bioactivity of SP, rather than a validated molecular pharmacological mechanism or a therapeutic application. Future studies are needed to clarify the precise molecular targets and translational potential of this marine polyphenol.

## 4. Conclusions

In this study, an ultrasound-assisted extraction process for *Sargassum fusiforme* polyphenols (SP) was established using response surface methodology. The optimal extraction parameters were identified as: ethanol concentration of 50%, liquid-to-solid ratio of 40 mL/g, ultrasonic power of 150 W, extraction temperature of 50 °C, and extraction time of 60 min. Under these conditions, the SP yield reached 38.91 ± 0.82 mg/g, with experimental values closely matching model predictions, validating the reliability of the established process. In functional assessments, SP demonstrated radical scavenging activity in cell-free systems and cytoprotective effects in H_2_O_2_-challenged and LPS-stimulated RAW 264.7 macrophages. At the cellular level, SP treatment was associated with increased activities of endogenous antioxidant enzymes (SOD, CAT and GSH-Px), alongside reduced levels of pro-inflammatory mediators (NO, TNF-α, IL-1β, IL-6 and PGE_2_) and an elevated level of IL-10. These observations correlated with changes in the expression of proteins involved in the Nrf2/ARE, MAPK and NF-κB signaling axes, suggesting potential regulatory interfaces rather than established causal relationships.

Several limitations of this study should be acknowledged. First, the experiments were conducted exclusively in vitro using a single macrophage cell line, and the chemical composition of SP was not systematically characterized, leaving the specific bioactive constituents unidentified. Second, the proposed involvement of Nrf2/ARE, MAPK, and NF-κB pathways was inferred from changes in protein expression levels, without direct evidence of molecular interactions such as binding affinity or genetic perturbation. Future investigations should prioritize comprehensive phytochemical profiling, in vivo efficacy and safety evaluations, and direct mechanistic validation using biophysical or genetic approaches. Additionally, the transferability of the established extraction protocol to pilot-scale or industrial settings remains to be determined. Collectively, this study provides a laboratory-scale extraction protocol for SP and presents in vitro evidence for its antioxidant and anti-inflammatory bioactivities, while clearly delineating the experimental boundaries of the current findings. The results serve as a preliminary experimental basis for further investigation into the bioactive potential of this marine polyphenol, rather than a validated therapeutic or industrial solution.

## Data Availability

The original contributions presented in this study are included in the article/Supplementary Materials. Further inquiries can be directed to the corresponding authors.

## Supplementary Materials

The following supporting information can be downloaded at: https://www.mdpi.com/article/10.3390/foods15162821/s1.
