# Supplementary material for: Process Optimization of Sargassum fusiforme Polyphenols and Their Cytoprotective Effects Against Oxidative and Inflammatory Injury, with Potential Signaling Correlates
Source: Foods. 2026 Aug 13;15(16):2821. doi: 10.3390/foods15162821 (PMC13511828; doi:10.3390/foods15162821)
Supplement: Supplementary file 1 [file foods-15-02821-s001.zip › foods-4449221-supplementary.pdf]

Table S1. Primer sequences for real-time reverse transcription-polymerase chain reaction (RT-PCR).

Primer		Primer sequence (5’–3’)
CAT	Forward	CCATTATAAGACTGACCAGGGC
	Reverse	AGTCCAGGAGGGGTACTTTCC
SOD-1	Forward	TGGTTTGCGTCGTAGTCTCC
	Reverse	CTTCGTCGCCATAACTCGCT
SOD-2	Forward	AGGACCTGCCTTACGACTATG
	Reverse	TGGACACTGCTCCTTGGTATT
GSH-Px	Forward	CAGTCGGTGTATGCCTTCTCG
	Reverse	GAGGGACGCCACATTCTCG
iNOS	Forward	CCCTTCCGAAGTTTCTGGCAGCAGC
	Reverse	GGCTGTCAGAGCCTCGTGGCTTTGG
COX-2	Forward	CACTACATCCTGACCCACTT
	Reverse	ATGCTCCTGCTTGAGTATGT
IL-1β	Forward	CAGGATGAGGACATGAGCACC
	Reverse	CTCTGCAGACTCAAACTCCAC
IL-6	Forward	GTACTCCAGAAGACCAGAGG
	Reverse	TGCTGGTGACAACCACGGCC
TNF-α	Forward	TTGACCTCAGCGCTGAGTTG
	Reverse	CCTGTAGCCCACGTCGTAGC
IL-10	Forward	GACTTTAAGGGTTACCTGGGTTG
	Reverse	TCACATGCGCCTTGATGTCT
β-actin	Forward	GTGGGCCGCCCTAGGCACCAG
	Reverse	GGAGGAAGAGGATGCGGCAGT

Table S2. Primer sequences for real-time reverse transcription-polymerase chain reaction (RT-PCR).

Primer		Primer sequence (5'–3')
Nrf2	Forward	TCACACGAGATGAGCTTAGGGCAA
	Reverse	TACAGTTCTGGGCGGCGACTTTAT
Keap1	Forward	CAGCAACTCTGTGACGTGACC
	Reverse	TCAATAAGCCTTTCCATGACC
NQO1	Forward	GGTGAGCTGAAGGACTCGAA
	Reverse	ACCACTGCAATGGGAACTGAA
HO1	Forward	ATGGCCTCCCTGTACCACATC
	Reverse	TGTTGCGCTCAATCTCCTCCT
ERK 1/2	Forward	CCAGGATTACCTGCCTCAAG
	Reverse	TCTGTAGCCACCACCAATCC
JNK	Forward	GAG AAG GAC TCC CAG CAT GA
	Reverse	GTC AGG GTA GGC AAG GAG TC
p38	Forward	TTC CGA TGG AAC TGT GAC CC
	Reverse	GTT GCT GGC AAT GAG CGA TA
MyD88	Forward	GGA ACT GCA GAC CTG GAA GG
	Reverse	GCT GGT GGA GTT CAA GTC GG
IκB-α	Forward	AGG CCA GGA GAC CAG GAA T
	Reverse	TCC TTG AGC TCA GGA ATC CG
p65	Forward	AGC ACC AGC TGG TCA GGT AT
	Reverse	TCT GGC TGG ACT CCA TGA TC
β-actin	Forward	GTGGGCCGCCCTAGGCACCAG
	Reverse	GGAGGAAGAGGATGCGGCAGT

Table S3. Response Surface Test Factor Levels.

Factors	Levels		
	-1	0	1
A: Ethanol concentration (%)	40.00	50.00	60.00
B: Liquid-to-solid ratio	35.00	40.00	45.00
C: Ultrasonic power (W)	120.00	150.00	180.00

Table S4. RSE Optimization for the SP.

Run	Factor 1<br>A: Ethanol concentration (%)	Factor 2<br>B: Liquid-to-solid ratio	Factor 3 C: Ultrasonic power (W)	Response 1<br>Content (mg/g)
1	50.00	45.00	120.00	33.73±2.87
2	60.00	45.00	150.00	35.35±2.57
3	50.00	40.00	150.00	39.98±1.89
4	50.00	40.00	150.00	39.23±1.22
5	50.00	40.00	150.00	39.93±1.67
6	50.00	40.00	150.00	37.32±1.62
7	50.00	45.00	180.00	35.22±2.61
8	50.00	35.00	180.00	32.71±2.64
9	50.00	40.00	150.00	38.21±2.11
10	40.00	40.00	120.00	30.23±1.62
11	60.00	35.00	150.00	33.18±2.76
12	40.00	35.00	150.00	32.33±2.31
13	40.00	40.00	180.00	32.19±2.15
14	60.00	40.00	180.00	30.81±2.46
15	60.00	40.00	120.00	31.78±2.74
16	50.00	35.00	120.00	31.42±1.27
17	40.00	45.00	150.00	32.81±1.87

Data are presented as mean ± standard deviation of triplicate experiments.

Table S5. ANOVA for the response surface quadratic model.

Source	Sum of Squares	df	Mean Square	F Value	p-value Prob > F	Significance
Model	222.77	9	24.75	37.83	< 0.0001	significant
A-A	1.58	1	1.58	2.42	0.1637	
B-B	6.98	1	6.98	10.66	0.0138	
C-C	1.78	1	1.78	2.72	0.1434	
AB	0.71	1	0.71	1.09	0.3309	
AC	2.15	1	2.15	3.28	0.1130	
BC	0.010	1	0.010	0.015	0.9051	
A^2	80.30	1	80.30	122.72	< 0.0001	
B^2	23.24	1	23.24	35.52	0.0006	
C^2	85.81	1	85.81	131.15	< 0.0001	
Residual	4.58	7	0.65			
Lack of Fit	2.29	3	0.76	1.33	0.3827	not significant
Pure Error	2.29	4	0.57			
Cor Total	227.35	16				

Table S6. LC-MS/MS identification of polyphenolic compounds in enriched SP fractions.

No.	Rt (min)	Formula	Quasi-molecular Ion (m/z)	Major fragments (one decimal)	Molecular species identification	Peak area (Mean value)
1	2.25	$C_8H_{14}O_3$	157	139, 113, 97	3-Oxooctanoic acid	3452271
2	2.78	$C_6H_6O_3$	125	97	Phloroglucinol	645281
3	3.22	$C_4H_8O_5$	135	117, 91	Threonic acid	3161896
4	4.23	$C_{18}H_{14}O_{10}$	389	125, 245, 265	Trifuhalol	328976
5	4.83	$C_{46}H_{40}O_{25}$	513	246, 265, 389	Tetrafuhalol	5682791
6	5.09	$C_{15}H_{10}O_4$	253	225, 197, 143	4’,7-Dihydroxyisoflavone	68326194
7	5.34	$C_{18}H_{36}O_2$	283	265, 237, 240	Stearic acid	21901644
8	5.54	$C_{12}H_{14}O_4$	221	193, 149	Diethyl phthalate	6318926
9	5.76	$C_{18}H_{12}O_{12}$	263	111, 219, 245	Carmalol	8752315
10	6.26	$C_{18}H_{12}O_9$	371	121, 140, 246	Eckol	53618862
11	6.56	$C_{20}H_{32}O_2$	303	259, 285,	Arachidonic acid	216186459
12	6.87	$C_{16}H_{16}O_6$	303	163, 271	3'-O-Methylcatechin	9361511
13	7.54	$C_{22}H_{44}O_2$	339	211, 295, 321	Behenic acid	765167

Table S7. DPPH and ABTS radical scavenging activities of SP and ascorbic acid at various concentrations.

Concentration (mg/mL)	DPPH Scavenging Rate (%)		ABTS Scavenging Rate (%)	
	SP Inhibition Rate (%)	Ascorbic Acid Inhibition Rate (%)	SP Inhibition Rate (%)	Ascorbic Acid Inhibition Rate (%)
0.0002	12.32± 1.82	15.62± 2.09	9.82± 1.51	12.32± 1.87
0.0005	22.51± 2.31	28.41± 2.69	18.62± 2.12	23.51± 2.39
0.001	36.72± 2.92	42.11± 3.21	31.22± 2.68	37.82± 3.01
0.002	50.21± 3.13	56.82± 3.52	48.51± 3.01	55.21± 3.28
0.004	68.41± 3.61	74.18± 3.81	65.72± 3.48	72.12± 3.68
0.008	82.12± 4.22	88.49± 4.01	79.32± 4.01	85.61± 4.11
0.016	91.52± 4.48	94.29± 4.29	89.41± 4.29	92.82± 4.51
IC_{50} (mg/mL)	0.00198	0.00145	0.00215	0.00162
95% Confidence Interval	0.00172–0.00228	0.00123–0.00171	0.00188–0.00246	0.00139–0.00189
R^2	0.994	0.997	0.992	0.995

IC_{50} values were calculated by nonlinear regression analysis using a four-parameter logistic model (log[concentration] vs. normalized response) with GraphPad Prism 9.0. CI, confidence interval.

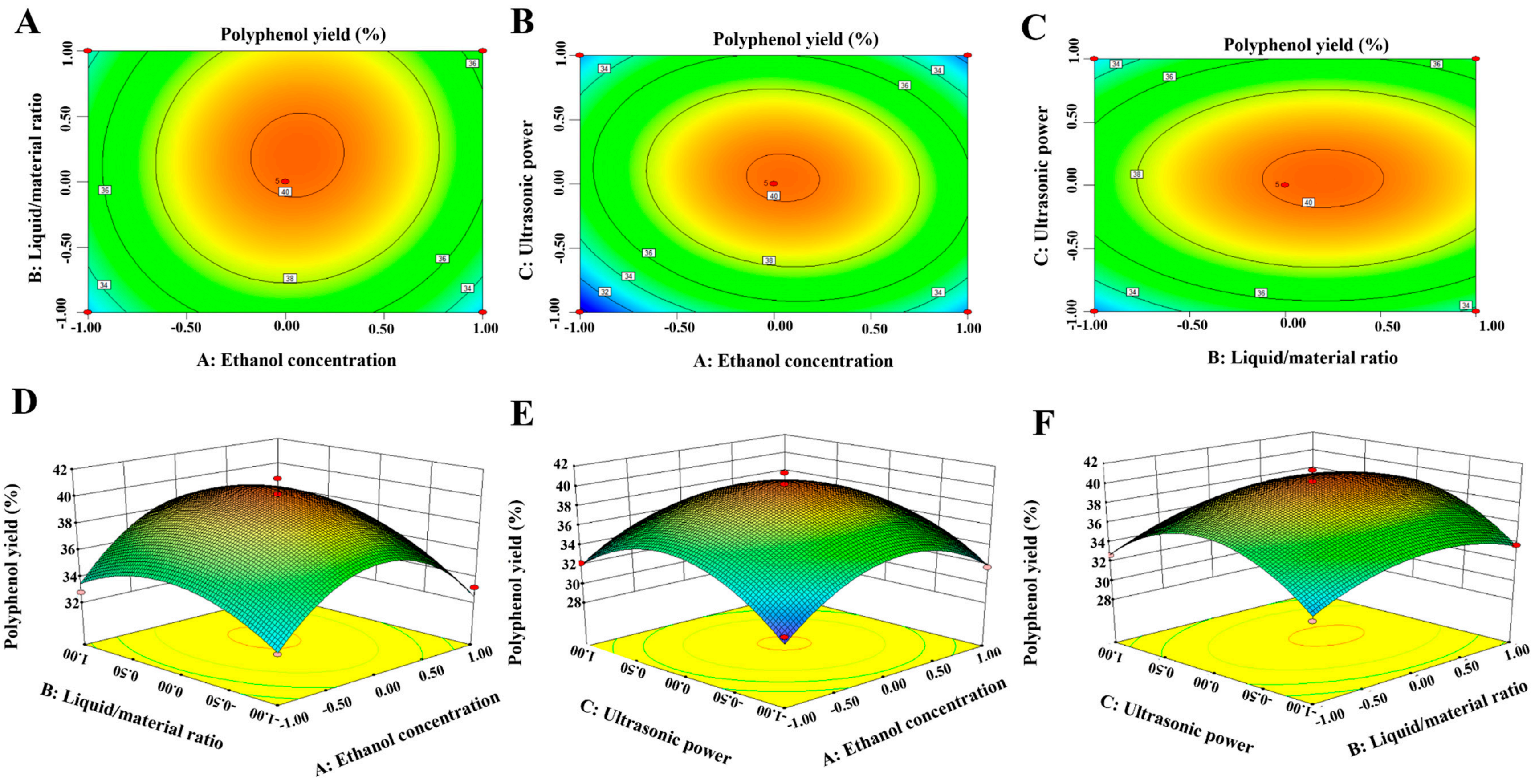

Figure S1: (A–F) Three-dimensional response surface plots showing the interactive effects of ethanol concentration (A), liquid-to-solid ratio (B), and ultrasonic power (C) on the extraction yield of SP.

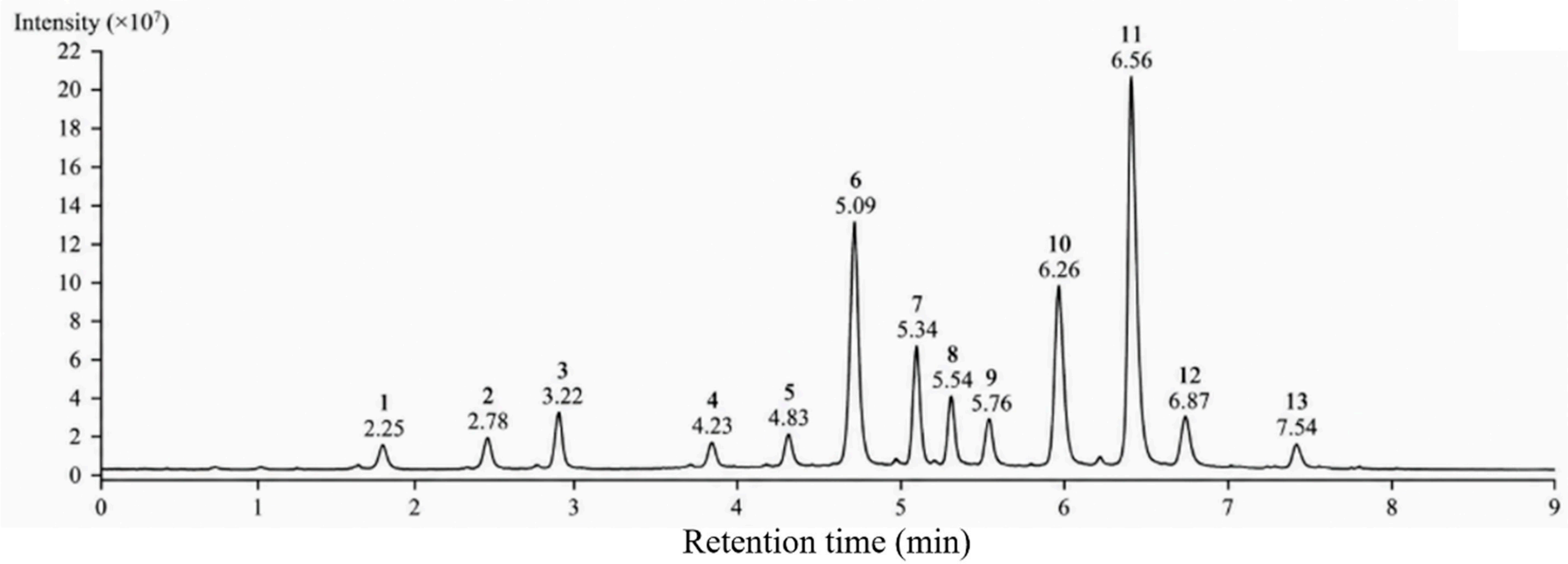

Figure S2: Total ion chromatograms (TIC) of SP fractions analysed by LC–MS/MS.

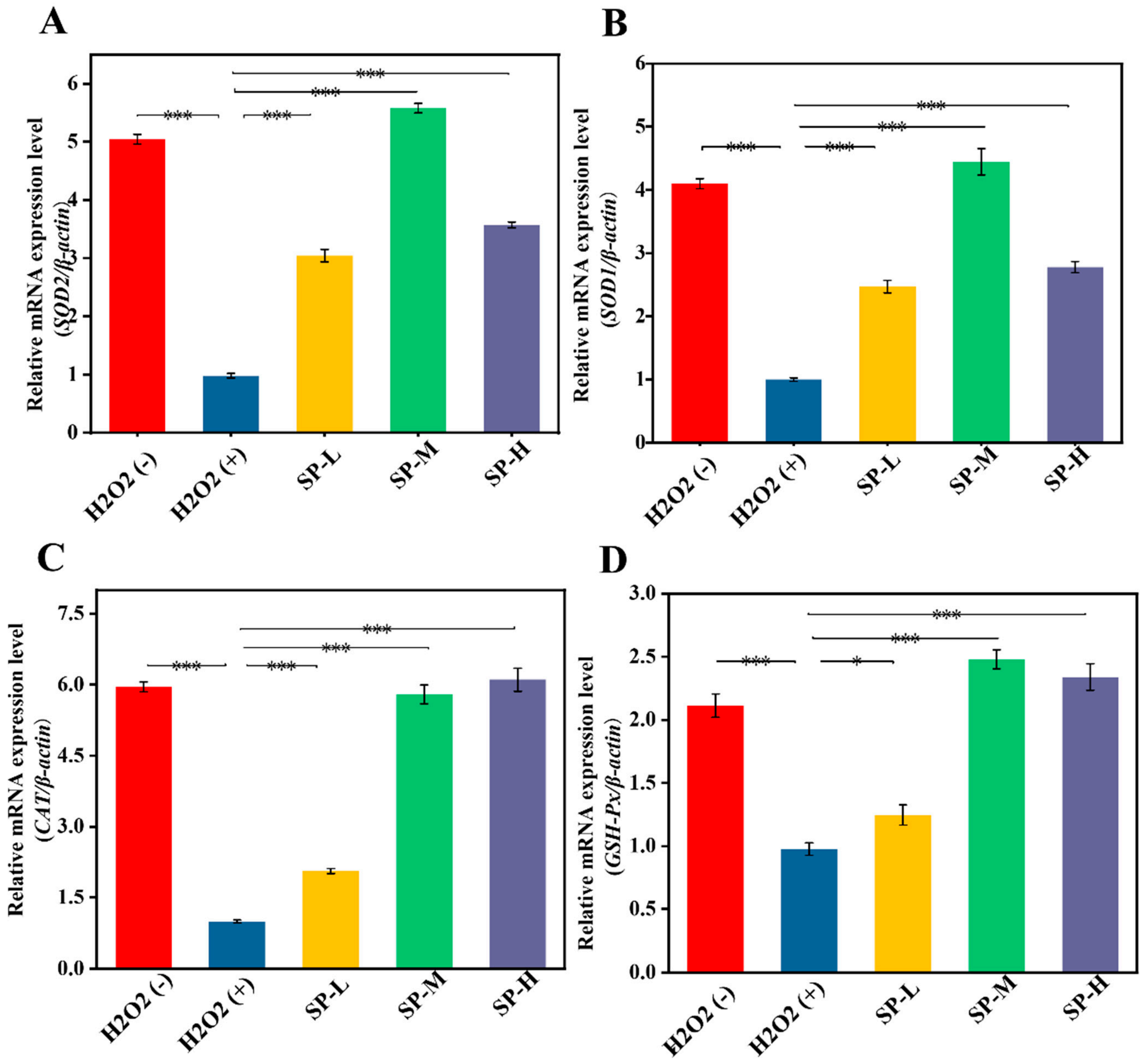

Figure S3: In vitro antioxidant capacity of SP in RAW 264.7 macrophages. Relative mRNA expression levels of (A) SOD2, (B) SOD1, (C) CAT, and (D) GSH-Px. $P < 0.05$ (*), and $P < 0.001$ (***).

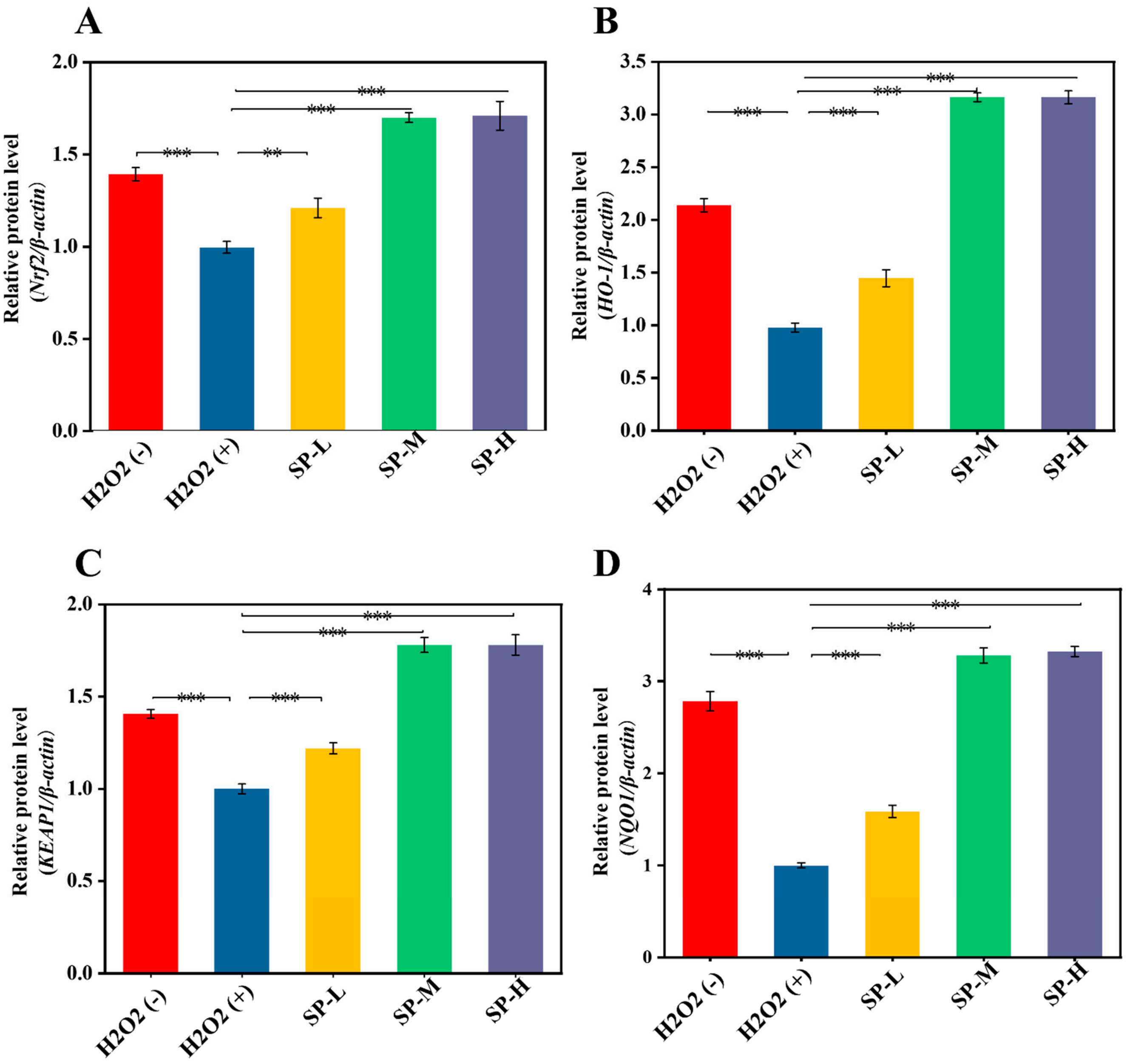

Figure S4**.** Effects of SP on the Nrf2/ARE signaling pathway in RAW 264.7 macrophages. Relative protein levels of (A) Nrf2, (B) HO-1, (C) KEAP1, and (D) NQO1. $P < 0.01$ (**), and $P < 0.001$ (***).

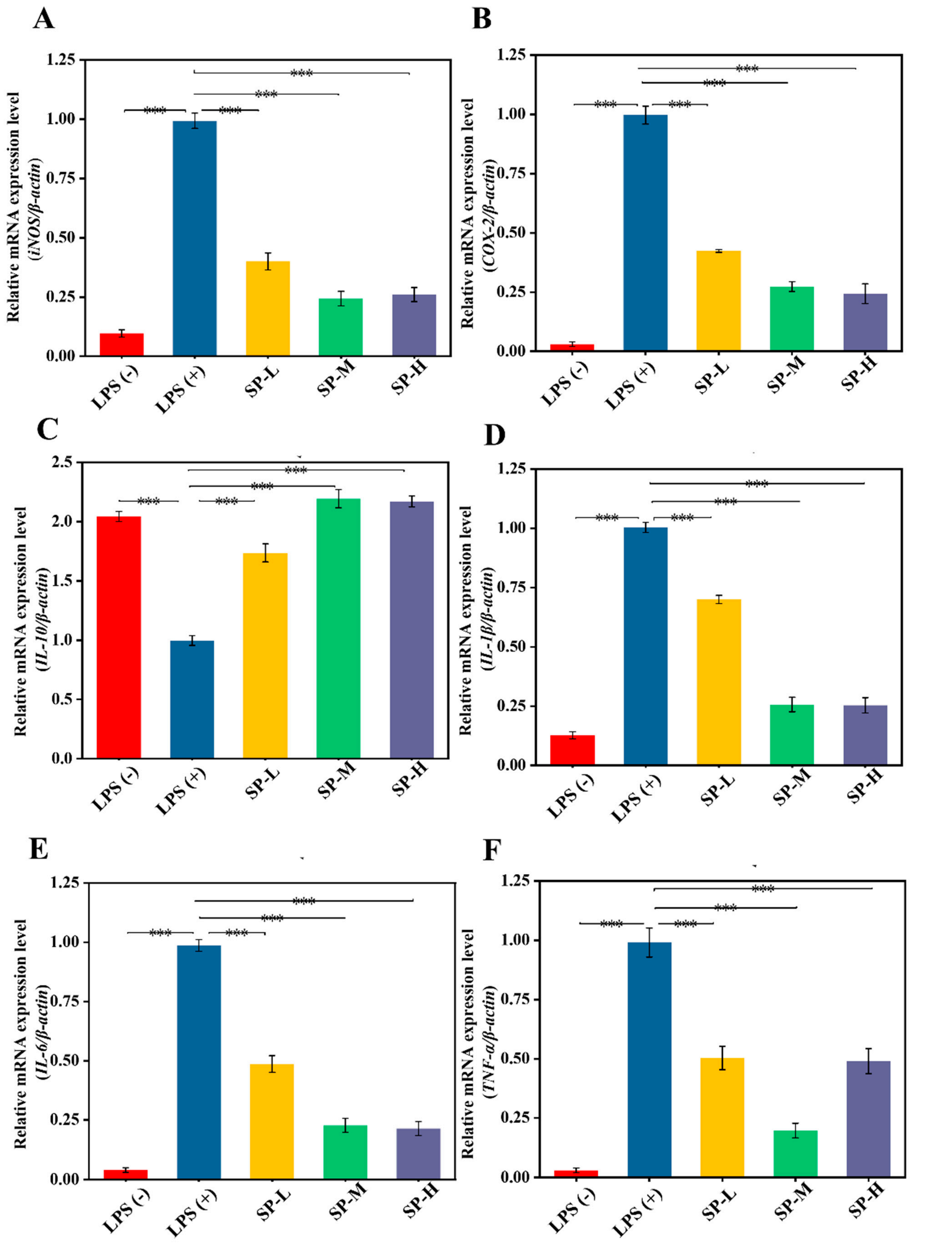

Figure S5. Relative mRNA expression levels of (A) iNOS, (B) COX-2, (C) IL-10, (D) IL-1β, (E) IL-6, and (F) TNF-α. $P < 0.001$ (***).

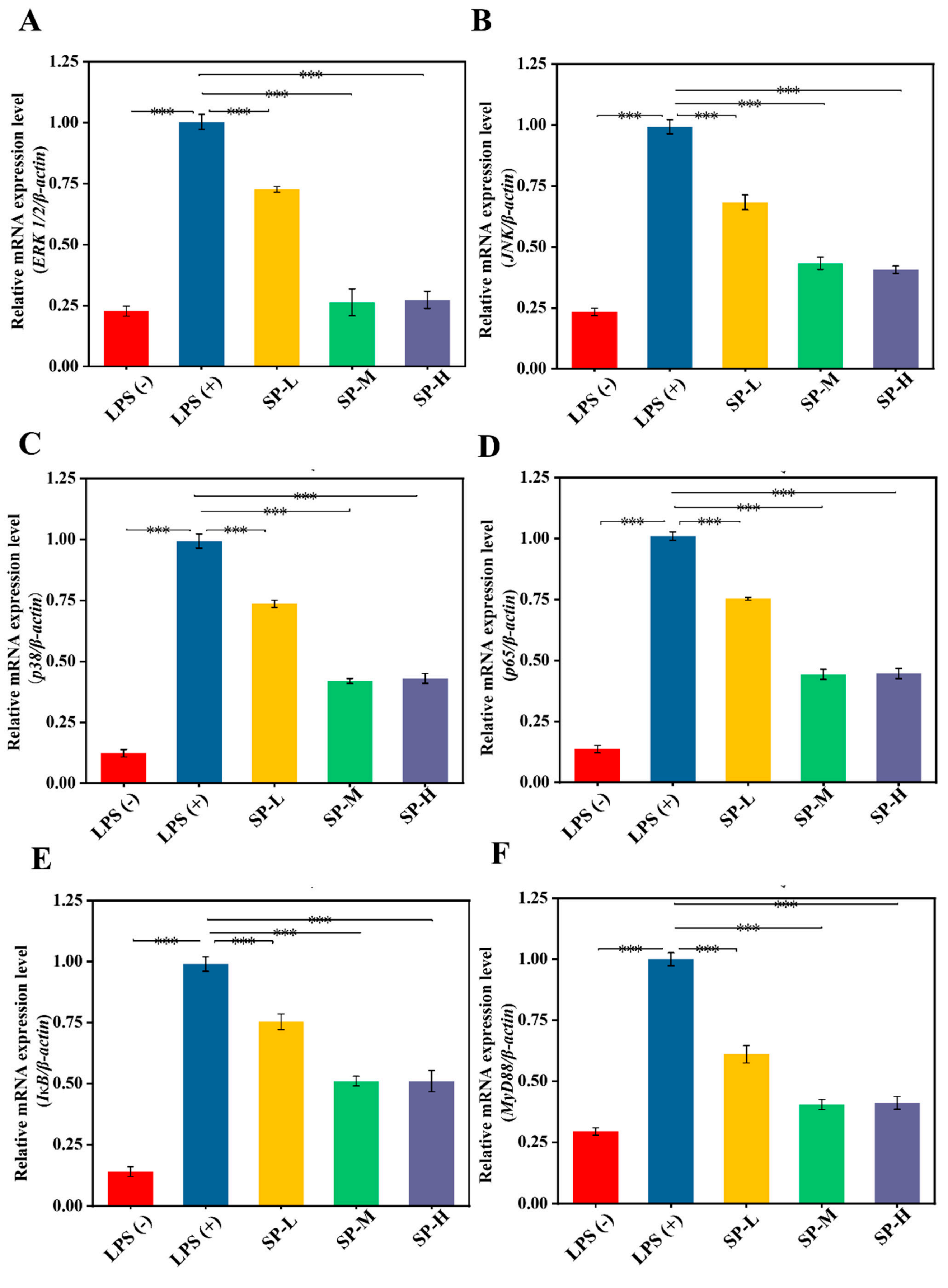

Figure S6. Effects of SP on LPS-induced activation of MAPK and MyD88/NF-κB signaling pathways in RAW 264.7 macrophages. The relative mRNA expression level of (A) ERK 1/2, (B) JNK, (C) p38, (D) p65, (E) IκB, and (F) MyD88. $P < 0.001$ (***).
